# 

**Published:** 1893-04-15

**Authors:** 


**OUR NEW OFFICES.**

428, Strand, W.C., will henceforth be the Office of this Journal.

**Notices of Births, Deaths, and Marriages,** are inserted in this column at a charge of 2s. 6d. for an announcement not exceeding four lines.

### NOTICE TO CORRESPONDENTS.

All MS., Letters, Books for Review, and other matters intended for the Editor should be addressed The Editor, The Lodge, Porchester Square, London, W.

The Editor cannot undertake to return rejected M.S., even when accompanied by stamped directed envelope.

**Notice to Advertisers and Subscribers.**

All Advertisements, Orders for Copies of The Hospital, and Business Communications should be addressed to The Publisher, *not to the Editor*, The Hospital, 428, Strand, W.C.
The Cost of Subscription, post free, is as follows:—Per week, 2½d.; per quarter, 2s. 6d.; per half-year, 5s.; per annum, 8s. 6d. Foreign:—Per annum, 10s. 8d.; payable, in all cases, in advance.

**"Burdett's Hospital Annual," 1893.**

Fourth year of publication. 700 pp. Boards, gilt lettering. A most complete guide to British, Colonial, Indian, and American Hospitals, Dispensaries, Nursing and Convalescent Institutions, and Asylums. Price 5s. Published by The Scientific Press (Limited), 428, Strand, W.C.

**NOTICE.—The Index for Vol. XII. (ending September 1892) is on sale at the Office of this Journal, price 2½d., post free.**

### Books Received.

T. and A. Constable, Edinburgh.

"Atlas of Clinical Medicine." Vol. II., Part II. By Dr. Byrom Bramwell. 1893.

**Periodicals and Pamphlets.**—G. Newnes: The Strand Magazine and The Picture Magazine. Cassell and Co.: Cassell's Family Magazine. S. W. Partridge and Co.: Onward and Upward. James Nisbet and Co.: The Indian Female Evangelist. E. Knight: The Clinical Journal. Chas. J. Clark: Oratava as a Health Resort. Longmans, Green, and Co.: The Charity Organisation Review. The National Temperance Publication Depot: The Medical Pioneer. E. Kirby: Work and Leisure. John F. Shaw and Co.: Service for the King. Horace Marshall and Son: London. E. W. L. Bellan: Leading Opinion. Kegan Paul, Trench, Trubner, and Co.: The Child's Guardian. Mecredy and Kyle: The Health Record. J. and A. Churchill: The Birmingham Medical Review

Toilers of the Deep, The Journal of Mental Science, and The Therapeutic Gazette.

### SCRAPS AND GLEANINGS.

The Hanley, Stoke, and Fenton Joint Hospital Board have purchased a new ambulance for £60.

The Duke of Cambridge has consented to preside over a dinner in aid of the funds for the Richmond Hospital.

The majority of cotton mills in Ashton under-Lyne district, after a stoppage of over three months, have resumed work.

The Bristol Hospital for Sick Women and Children reports a balance of £37 4s. to the good at this year's annual meeting.

Swansea Hospital is trying to increase its funds, and increase the interest and support of the working classes towards it.

The Fever Hospital at Hither Green, Lewisham, may not, after all, be erected on the site selected by the Asylum authorities.

Some new bridges are contemplated for Pittsburg, a city which already possesses no fewer than 14, spanning the rivers Monongahola and Alleghany.

A negro was lately executed by electricity in Sing Sing Prison, New York. The strength of the current, which was applied for one minute, was 1,740 volts.

The Liberator convicts (Hobbs, Newman, and Wright) are removed to Wormwood Scrubbs, where the first nine months of their imprisonment will be passed.

The Duke of Connaught has consented to give his patronage to the ball at the Hotel Metropole on May 2nd, which is to be held in aid of the Home of Rest for Nurses.

The Royal National Hospital for Consumption at Ventnor has received a gift of £1,000 from Mr. F. C. Nunn and Mr. P. J. Nunn, in memory of their late father.

The yearly manufacture of boots in this country is estimated at 49,736,000 pairs—valuing more than £12,000,000—a large proportion of which find their way to the Colonies.

The concert given at the Town Hall, Folkestone, in aid of the Sandgate Landslip Relief Fund, proved a financial success. The Duke of Westminster has forwarded £20 to the fund.

Mr. G. Corbett, of Droitwich, has made a special donation of £20 to the Margaret Street Hospital for Consumption and Diseases of the Chest, of which institution Mr. Corbett is a patron.

The Committee of the Liverpool Hospital for Cancer and Skin Diseases reports that the returns of the out-patients show 31,17[illegible] attendances, giving an average of 102 attendances the day.

London is the largest brewing centre in England, consuming 10,956,363 bushels of malt and corn, and having 139 breweries. Burton comes next with a consumption of 6,228,906 bushels.

The Birmingham and Midland Counties Training Institution for Nurses report the number of 82 on their books and 13 probationers, making a total of 95. 752 families have been attended.

The leading spirits among the Russian Nihilists propose coming together at Whitsuntide at some town in South Russia to discuss the most pressing questions of the day as affecting their party.

The Birmingham and Midland Hospital for Women have treated 13,711 cases during the year. The alterations which had lately been effected at Sparkhill exceeded the amount raised by about £250.

The report of the York County Hospital is a very satisfactory one. The Committee declared its present position, financially and medically, to be a good one. Donations to the hospital during the year amounted to £1,031.

It is proposed to erect a Medical Club Building at Detroit, there being five Medical Societies and 441 physicians in that city, and a united building for common scientific and social purposes is suggested accordingly.

The Royal Portsmouth, Portsea, Gosport Hospital reports a satisfactory financial outlook. The hospital has benefited by several legacies and donations, the latter including £1,000 from Mr. T. S. Edgcombe.

Sir Charles and Lady Halle have kindly promised their services at the concert which will be given by the Stock Exchange Orchestral Society at St. James's Hall, on the 9th prox., in aid of the Chelsea Hospital for Women.

In connection with the forthcoming festival of the Royal Hospital for Diseases of the Chest, Mr. De La Rue has promised a donation of three hundred guineas, and a hundred guineas on behalf of his firm, Messrs. Thomas De La Rue and Co.

It is gratifying to observe an increase in the annual subscriptions and church collections for the Paisley Infirmary, and that the balance at the debit of the revenue account existing at the beginning of the year has, so far, been reduced.

At a post mortem examination of a female lunatic in a Russian lunatic asylum, the doctors discovered three teaspoons, a piece of iron, and two triangular pieces of glass. Her death was not caused by these foreign bodies, but was the result of disease of the brain.

A great falling off in annual subscriptions is reported from the Loughborough General Hospital. From the year 1880 the subscriptions had gradually fallen, until they now stood at £287. A personal and direct appeal for the increase of funds is to be made shortly.

The Manchester Hospital for Consumption to be placed on a sound basis requires an additional income of about £500 per annum. Unless the appeal for increased funds is largely responded to there is a danger that the board will be compelled to limit the number of patients.

The Committee of the Bristol Hospital for Sick Women and Children, at the annual meeting, alluded with satisfaction to the fact that the out-patient department was partially self-supporting, the expenditure thereon being £59[illegible], but towards that £4 0 had been paid by the patients.

A two days' bazaar, in aid of the funds of the Blackburn Orphanage, which was held last month in the Exchange Hall, the receipts amounting to over £700, Mr. Tattershall forwarded a further cheque of £50. To meet the necessity of immediate repairs to the orphanage £4 0 is still necessary.

For the Book World of Medicine and Science, and Student of Medicine, and Medical Appointments, see pages VIII., IX., X., and XI. overleaf.

### FIRST IMPRESSIONS OF BOOKS RECEIVED.

[It is requested that all books intended to be noticed in this column may be sent to the Editor, at the Office, 428, Strand, W.C., not later than Tuesday evening in each week.]

The Scalpel. A Monthly Magazine for Medical Students. March, 1893.

Our Indian medical students are well abreast of the times, and this little periodical is issued in Calcutta by and for them. The number before us contains medical, athletic, social, and comic articles, such as are dear to the hearts of medical students in all lands.

Practical Pharmacy for Medical Students. By A. Campbell Stock, Demonstrator of Materia Medica and Pharmacy at St. George's Hospital. London: Baillière, Tindall, and Cox, 1893.

This is a little book of 200 pages, easily carried in the pocket. It is specially designed for the use of students preparing for the examination in Practical Pharmacy of the Conjoint Board of England. It will probably be popular with students, as it gives the information they want concisely and accurately, and its scope is limited by the requirements of the examination.

### THE STUDENT OF MEDICINE.

[All communications for this Supplement should be addressed to THE EDITOR of THE HOSPITAL, at 428, Strand, with the words, "Students' Column," written in the left hand top corner of the envelope.]

### APPOINTMENTS.

S. H. Adams, M.D.Lond., M.R.C.S.Eng., appointed Medical Officer for the Bedford and Kempton Sanitary Districts of the Bedford Union. H. W. Armstead, M.R.C.S., L.R.C.P., appointed a Senior House Surgeon to St. Bartholomew's Hospital. G. P. Barton, M.R.C.S.Eng., appointed Medical Officer for the Sub-District of Morchard Bishop of the Crediton Union. C. E. Bashall, L.R.C.P., M.R.C.S., appointed Assistant House Surgeon to the South Devon and East Cornwall Hospital, Plymouth. Douglas J. M. Bone, M.B., C.M.Edin., appointed Medical Officer of Health for the Lunesdale Rural Sanitary District. E. C. Bridges, M.R.C.S., L.R.C.P., appointed a Junior House Physician to St. Bartholomew's Hospital. R. Brown, M.R.C.S., L.R.C.P., appointed Extern Midwifery Assistant to St. Bartholomew's Hospital. A. H. Buck, M.R.C.S., L.R.C.P., appointed a Junior House Surgeon to St. Bartholomew's Hospital. C. Buttar, M.B.Cantab., appointed a Junior House Surgeon to St. Bartholomew's Hospital. J. Chadwick, M.R.C.S.Eng., L.S.A., appointed Medical Officer for the Butterworth District of the Rochdale Union. Dr. Clegg, reappointed Medical Officer for the Whitworth District of the Rochdale Union.

### VACANCIES.

The following vacancies are announced this week, the amount of salary in each case being given after the name of the institution:

### Resident Medical Officers.

Birmingham General Dispensary, Birmingham. Resident Surgeon for the Highgate Branch; doubly qualified. Salary £150 per annum, with allowance for cab hire, and furnished rooms, fire, lights, and attendance. Applications to Alex. Forrest, Secretary, by May 17th.

### MEDICAL APPOINTMENTS.

### ROYAL FREE HOSPITAL, GRAY'S INN ROAD.

### LIVERPOOL STANLEY HOSPITAL.

### CITY OF LONDON HOSPITAL FOR DISEASES OF THE CHEST, Victoria Park, E.

Applications, with testimonials, for the POST of PATHOLOGIST, are invited to be sent to the SECRETARY, at the Office, 24, Finsbury Circus, E.C., not later than April 20th.

Candidates must be registered and not engaged in General Practice.

Salary 100 guineas per annum.

Information as to duties, &c., can be obtained from the Secretary on application by letter or personally at the Office any day between Twelve and Two o'clock.

(261) T. STORRAR SMITH, Secretary.

### BIRMINGHAM GENERAL DISPENSARY.

### THE STUDENT OF MEDICINE—(*continued*).

Bath General or Royal Mineral Water Hospital, Bath. Resident Medical Officer; unmarried. Salary £100 per annum, with board and apartments. Applications to Fred. W. Dingle, Registrar and Secretary, by April 17th.

County Asylum, Rainhill, near Liverpool. Assistant Medical Officer, unmarried, and not more than 30 years of age. Salary to commence at £100 per annum, with prospect of £25 increase at the end of first and second years, and with further increase according to promotion, together with furnished apartments, board, and attendance. Applications to the Medical Superintendent.

County Asylums, Gloucester. Third Assistant Medical Officer, doubly qualified, unmarried, and not more than 30 years of age. Salary £105 per annum, with board, lodging, and washing. Applications to the Medical Superintendent by April 18th.

Miller Hospital and Royal Kent Dispensary, Greenwich Road, S.E. Junior Resident Medical Officer. Salary £30 per annum, with board, attendance, and washing. Applications to the Honorary Secretary by April 15th.

Royal Free Hospital, Gray's Inn Road. Senior Resident Medical Officer; doubly qualified. Salary £100 per annum, with board, residence, and washing. Also Junior Resident Medical Officer. Board, residence, and washing provided. Applications to Conrad W. Thies, Secretary, by April 17th.

St. Pancras and Northern Dispensary, 126, Euston Road. Resident Medical Officer; doubly qualified. Salary £105 per annum, with residence and attendance. Applications to the Honorary Secretary, H. P. Bodkin, 23, Gordon Street, Gordon Square, W.C., by April 20th.

The Coppice, Nottingham. Assistant Medical Officer; unmarried, not more than 28 years of age. Salary £120 per annum, with furnished apartments, board, washing, and attendance. Applications to Dr. Tate, at the Asylum, by April 24th.

### Non-Resident Medical Officers.

City of London Hospital for Diseases of the Chest, Victoria Park, E. Pathologist. Salary 100 guineas per annum. Applications to the Secretary, at the office, 24, Finsbury Circus, E.C., by April 20th.

Hastings, St. Leodards, and East Sussex Hospital, Hastings. Third Assistant Surgeon. Applications to the Secretary, by April 22nd.

Liverpool Stanley Hospital. Assistant Honorary Physician. Applications to the Honorary Secretary by April 20th.

Mason College, Birmingham. Co-Professor of Surgery. Applications to G. H. Morley by April 15th.

Plymouth Royal Eye Infirmary. Honarary Surgeon. Applications to the Secretary by April 15th.

